# Investigations on Structural, Optical and X-Radiation Responsive Properties of a-Se Thin Films Fabricated by Thermal Evaporation Method at Low Vacuum Degree

**DOI:** 10.3390/ma11030368

**Published:** 2018-03-02

**Authors:** Jitao Li, Xinghua Zhu, Dingyu Yang, Peng Gu, Haihua Wu

**Affiliations:** 1 College of Optoelectronic Technology, Chengdu University of Information Technology, Chengdu 610225, China; jtlee@foxmail.com (J.L.); xinghuazhu@cuit.edu.cn (X.Z.); gupeng.cuit@foxmail.com (P.G.); zhouyanzhenzhen@foxmail.com (H.W.); 2 College of Intelligent Manufacturing, Sichuan University of Arts and Science, Dazhou 635000, China

**Keywords:** amorphous selenium thin films, structure, optical properties, X-radiation response, thermal evaporation, low vacuum degree

## Abstract

Amorphous selenium (a-Se) thin films with a thickness of 1200 nm were successfully fabricated by thermal evaporation at a low vacuum degree of 10^−2^ Pa. The structural properties involving phase and morphology showed that a-Se thin films could be resistant to 60 °C in air. Also, a transformation to polycrystalline Selenium (p-Se) was shown as the annealing temperature rose to 62 °C and 65 °C, with obvious changes in color and surface morphology. Moreover, as the a-Se transformed to p-Se, the samples’ transmittance decreased significantly, and the band gap declined dramatically from 2.15 eV to 1.92 eV. Finally, the X-radiation response of a-Se was investigated as an important property, revealing there is a remarkable response speed of photogeneration current both X-ray on and X-ray off, with a requirement of only a very small electrical field.

## 1. Introduction

Amorphous selenium (a-Se) has been widely used for X-ray optoelectronic devices due to its excellent X-radiation responsive properties [1,2,3]. The thermal evaporation method is a good way to prepare a-Se films [4,5,6], and the background vacuum operating 10^−3^ Pa to 10^−4^ Pa is a current preparation condition [7,8,9]. However, it is well known that a high vacuum degree implies expensive equipment requirements and lots of vacuum preparation time, which apparently increase the production costs. By comparison, the vacuum degree of 10^−2^ Pa is very easy and quick to be achieved for any vacuum equipment. On the other hand, several reports have preferred to investigate the a-Se thick films with a thickness of tens of microns to hundreds of microns [4,5,6,7,10], since a-Se thick films can absorb more X-ray energy and thus produce a higher photocurrent; further, the systematic research on the a-Se thin films with the thickness of a few microns is rare [8]. Nevertheless, the low crystallization temperature, 40–50 °C, is a fatal disadvantage of a-Se thick films, as it seriously restricts its practical applications [11,12]. Theoretically, a-Se thin films possess the higher crystallization temperature, resulted from that the astrict of substrate hinders the migrations of atoms. Based on above considerations, we tried to deposit a-Se thin films at a low vacuum degree 10^−2^ Pa in order to save costs and improve the crystallization temperature, and new process parameters were explored in this work. Also, its structural, optical and X-radiation responsive properties were investigated, which revealed a potential application value for X-ray optoelectronic devices.

## 2. Material and Methods

**Figure 1:**
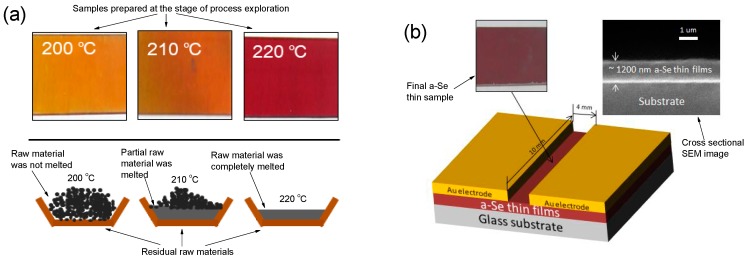

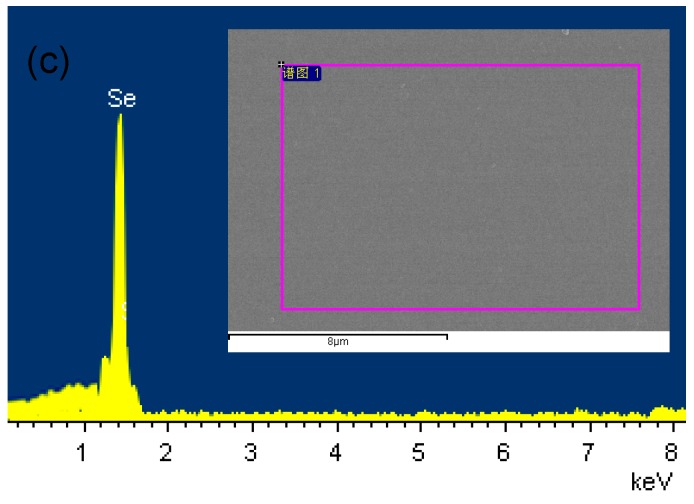
(**a**) The samples prepared at exploration process and their residual raw material; (**b**) the final amorphous selenium (a-Se) thin films sample with its sectional SEM image and brief structure; (**c**) Energy dispersive spectroscopy (EDS) image of the as-deposited sample.

Powder Se with a purity of 99.99% was used as a raw material and deposited on a glass substrate 30 cm distant to the evaporation source. The substrate temperature was room temperature (RT) 20 °C, and a metal evaporation dish with molybdenum (Mo) was used. Before deposition, the inner wall of the vacuum chamber was repeatedly cleaned using acetone and anhydrous ethanol, and the background vacuum pressure in the clean chamber was set at 10^−2^ Pa. Based on the lower vacuum degree than the previous reports, other parameters such as evaporation temperature had to be explored independently because of the lack of the related literatures. Generally speaking, powder Se should first be melted, and then evaporated to deposit a-Se thin films. In order to obtain the proper evaporation temperature of the raw material, we first deposited these samples at an evaporation temperature of 200 °C for 10 min, as shown in Figure 1a. However, the raw material was almost not melted, and a-Se thin films presented a canary yellow color, suggesting that the thickness of the films was so low that the evaporation temperature had to be enhanced. Further, the films’ thickness only slightly increased with the melting partially raw material at 210 °C. A sufficient deposition thickness was presented at 220 °C for 10 min, and the a-Se thin films clearly showed a change in color to magenta. Also, the powder was completely melted, illustrating that the 220 °C was an appropriate temperature to deposit a-Se thin films. Based on above explorations, three temperature stages were set to prepare the final sample, as shown in Figure 1b, including 200 °C for 10 min to evenly heat powder Se, 210 °C for 20 min to completely melt the powder, and then 220 °C for 45 min to deposit a-Se thin films with enough thickness. The thickness of the sample was approximately 1200 nm (Figure 1b), and was observed by scanning electron microscope (SEM, Hitachi S-4800, Tokyo, Japan) images. Meanwhile, considering the possible existence of some impurities from the atmosphere at low vacuum quality, the composition of the as-deposited sample was characterized by employing an energy dispersive spectroscopy (EDS) instrument integrated into a SEM instrument. Fortunately, the unique peak from Figure 1c confirmed the Se component without other components, preliminarily demonstrating that this vacuum quality was feasible for depositing pure a-Se thin films. In order to explore the crystallization temperature, the sample was divided into several parts to be annealed at 40 °C, 50 °C, 60 °C, 62 °C, and 65 °C in air, respectively. Besides, Au as electrode material was deposit on the surface of a-Se thin films to conveniently measure its X-ray response property using a precision source/measure unit (Agilent B2912A, Palo Alto, CA, USA) integrated an X-ray source (Oxford Instruments, Optima 97008, Oxford, Britain). X-ray diffraction (XRD, Fangyuan DX-2700, Dandong, China) patterns were investigated to characterize the phase. The surface morphology of the samples was observed by atomic force microscope (AFM, Benyuan BY-3000, Guangzhou, China), and the transmittance curves were obtained via Shimadzu UV-2550 instrument from Tokyo of Japan.

## 3. Results and Discussion

**Figure 2:**
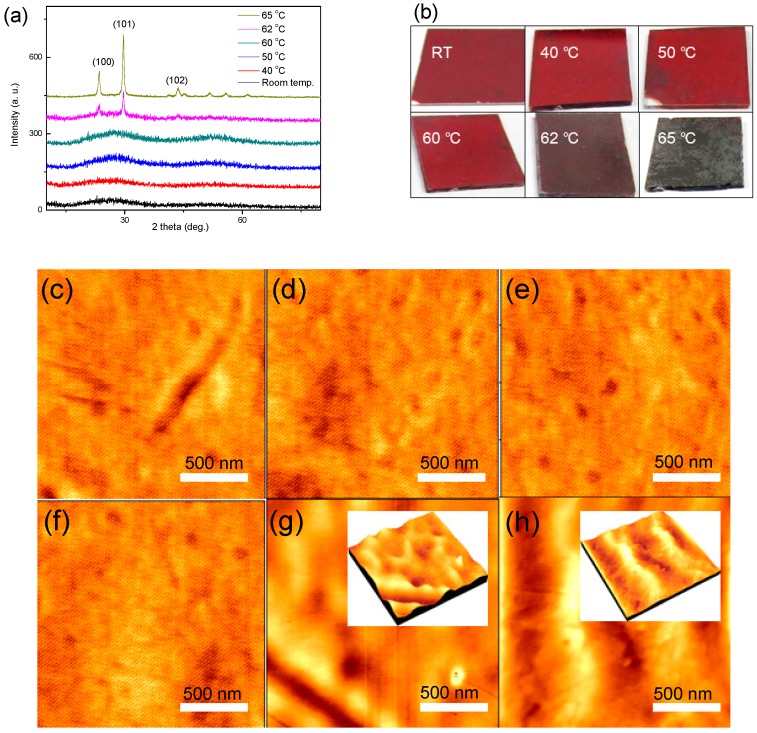
(**a**) XRD patterns of samples at different annealing temperature with (**b**) their practicality photographs; (**c**–**h**) Atomic force microscope (AFM) images of samples at different annealing temperature, (**c**) room temperature 20 °C, (**d**) 40 °C, (**e**) 50 °C, (**f**) 60 °C, (**g**) 62 °C, and (**h**) 65 °C.

Figure 2a shows the XRD patterns of samples that correspond to their practicality photographs in Figure 2b. The XRD patterns show the amorphous phase of samples below annealing 60 °C with their almost invariable practicality color. Next, a crystallization transition displays the various diffraction peaks in XRD patterns after annealing at 62 °C, corresponding to the darker color shown in practicality photographs. The darker color property of the polycrystalline Se (p-Se) is due to the strong optical absorptions in the visible range of long wavelengths [13], which will also be evidenced in the transmittance analysis section. It can be seen that the crystallization temperature at 62 °C for a-Se thin films is higher than for those of a-Se thick films [11,12], which is attributed to the significant influence from substrate surface. Specifically, these Se atoms on the substrate surface are hard to migrate to grow into a crystal core, because of the bondage of the substrate surface, which will exert more serious effects on the thinner a-Se films than the thicker a-Se films. As a result, the a-Se thin films have the higher crystallization temperature. Moreover, AFM images (Figure 2c–f) of a-Se thin films show that the non-crystalline surface morphology among different samples has almost no differences. Further, the AFM images (Figure 2g–h) of p-Se indicate the obvious particle shape, and the particle size increases gradually as the annealing temperature rises to 65 °C, suggesting the severer crystallization. The AFM images are in well accord with the results of the XRD patterns and practicality photographs.

**Figure 3:**
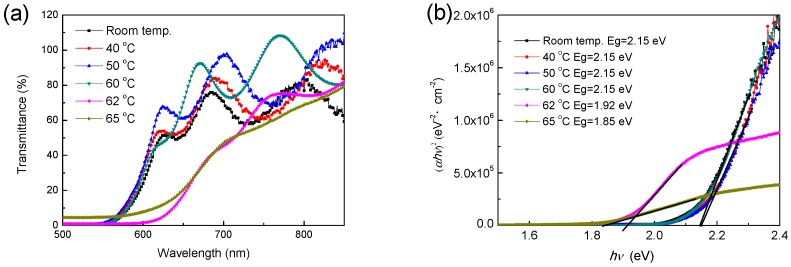
(**a**) Transmittance curves of samples at different annealing temperatures and (**b**) their energy gap curves.

As shown in Figure 3a, the transmittance of samples at non-crystalline state increases only slightly with annealing temperature attributed to the lower defect absorptions—the higher temperature provide the more energy to adjust the internal structure of thin films, reducing the defects concentrations. Comparing with the transmittance values of a-Se thin films in the visible long wavelength region, the transmittance of p-Se sees a dramatic decline, which causes the darker color as a result. Furthermore, it is an important distinction for a-Se and p-Se that the significant absorptions are respectively located at ~600 nm and ~680 nm in the transmittance spectra [14], which is assigned to the different optical band gap *Eg*. The band gap curves are shown in Figure 3b and deduced by Formulas (1) and (2):

(1) $$ahv^{2}=B\left(hv-Eg\right)$$

(2) a=−1/d×ln(T)

where *a* is the absorption coefficient related to thickness *d* of thin films and transmittance *T*, *hv* belongs to photon energy, and *B* is a constant. The *Eg* values of all the a-Se thin film samples are 2.15 eV, which agrees with the previous reports [15,16], and the *Eg* values of p-Se decrease from 1.92 eV to 1.85 eV with the annealing temperature up to 65 °C; apparently, the energy gap of a-Se is larger than that of p-Se. It can be considered that the amorphous phase is componented by the grains with very small size, thus this decrease in energy gap of samples with the transition from a-Se to p-Se can be attributed to quantum confinement effect (QCE) [17].

**Figure 4:**
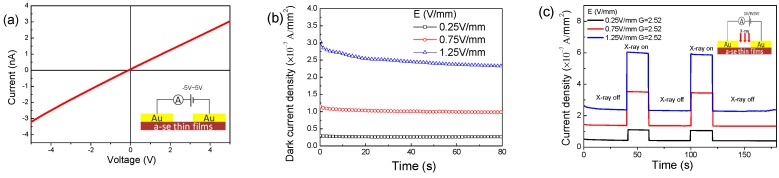
(**a**) I–V curves of a-Se thin films prepared at room temperature; (**b**) its dark current density and (**c**) X-ray photocurrent curves.

As a crucial feature, X-radiation responsive property as a function of both time and electrical field of a-Se thin films prepared at room temperature was investigated as shown in Figure 4. From Figure 4a, the linear I–V curve illustrates a good ohmic contact between Au electrode and a-Se films, which is a key prerequisite for testing accurately the dark current and X-ray photocurrent [18]. The dark current density measured at different bias voltage remains a 10^−7^ A/mm^2^ order of magnitude (Figure 4b), although the dark current rises with increasing bias voltage. Further, X-radiation responsive characterization with the current gain (G) of 2.52× of sample appears at weak electrical field (0.25 V/mm–1.25 V/mm), and the strong electrical field is not required by a-Se thin films comparing with driving electrical filed (tens of volts per micron) of a-Se thick films [2,4,5,7,8]. It can be seen from Figure 4c that the photogeneration current of a-Se thin films has a rapid response without obvious tailing both X-ray on and X-ray off, suggesting the low trap effects and high deposition quality of thin films [19]. X-ray response feature of a-Se thin films indicated a potential application for X-ray optoelectronic devices.

## 4. Conclusions

In this work, the a-Se thin films with a thickness of 1200 nm were successfully fabricated by thermal evaporation at low vacuum degree of 10^−2^ Pa. The a-Se thin films were able to resist the temperature as high as 60 °C in air. Comparing a-Se thin films at different annealing temperature, their practicality color, surface morphology, transmittance and energy gap (2.15 eV) were not almost changed. However, as annealing temperature up to 62 °C and 65 °C, a-Se transform to p-Se, AFM images showed obvious particle distribution as well as the darker practicality color resulted from the significantly improved absorptions in range of long wavelength, meanwhile, the band gap values (1.85–1.92 eV) of p-Se thin films were far lower than that of a-Se thin films because of QCE. Finally, benefited from the excellent deposition quality of a-Se thin films, X-radiation responsive property revealed that a requirement of small electrical field can achieve the rapid response of photogeneration current. Overall, this work provided a meaningful reference for fabricating the low cost thermal evaporation a-Se thin films at low vacuum degree.
